# Monkeypox Diagnosis by Cutaneous and Mucosal Findings

**DOI:** 10.3390/idr14050077

**Published:** 2022-09-27

**Authors:** Diogo de Sousa, João Patrocínio, Joana Frade, Claúdia Brazão, Dora Mancha, Catarina Correia, João Borges-Costa, Paulo Filipe

**Affiliations:** 1 Dermatology Department, Centro Hospitalar Universitário Lisboa Norte, 1169-050 Lisbon, Portugal; 2 Dermatology University Clinic, Faculty of Medicine, University of Lisbon, 1169-050 Lisbon, Portugal; 3 Instituto de Higiene e Medicina Tropical, Nova University of Lisbon, 1349-008 Lisbon, Portugal; 4 Dermatology Research Unit, Instituto de Medicina Molecular, University of Lisbon, 1169-050 Lisbon, Portugal

**Keywords:** monkeypox, skin, epidemiology, disease outbreak

## Abstract

A monkeypox outbreak has been reported in several countries since early May 2022. Human monkeypox (MPX) diagnosis is based on a clinical suspicion supported by typical skin and mucosal lesions, confirmed with molecular testing. We present the results of all MPX confirmed patients presenting to our department until July 15 of 2022, describing the characteristics of the lesions at diagnosis. In total, 47 patients were included, all men and 44.7% (*n* = 21) were HIV-positive. Skin lesions were noted in all patients. The most commonly affected area was the genital region (63.8%), followed by the anorectal region (46.8%). Extra anogenital mucosal (oral or conjunctival mucosa) involvement was reported in three patients. Typical skin findings included erythematous papules, whitish, umbilicated papules, some with a necrotic center and an elevated whitish border. Most patients had lesions in multiple phases presenting simultaneously. Correct identification of MPX skin and mucosal lesions is crucial to avoid late diagnosis and prevent further spreading, ensuring less worldwide morbidity.

## 1. Introduction

Human monkeypox (MPX) is a zoonotic disease caused by the monkeypox virus (MPXV), an orthopox DNA virus related to the virus that causes smallpox. MPX is an endemic pathogen among several African countries, where most outbreaks are linked to contact with wildlife reservoirs (mainly rodents) [1,2]. The infrequently reported outbreaks in non-endemic areas usually do not extend beyond a few transmission cycles, and person-to-person transmission was rarely described [3,4].

Since early May 2022, more than 27,000 MPX cases and 11 deaths have been reported in more than 89 countries, recently inciting the World Health Organization to declare monkeypox a “Public Health Emergency of International Concern” [5,6]. Since 3 May 2022, Portuguese public health authorities have documented several cases of MPX with sustained local human-to-human transmission, summing a total of 512 cases of confirmed MPX up to 14 July 2022, with 47 cases diagnosed and managed by our center [7].

MPX clinical syndrome is commonly characterized by fever, rash or skin lesions, and lymphadenopathy [8,9]. Most patients have a moderate form of the disease with no need for hospitalization or antiviral treatment; however, described MPX complications include pneumonitis, encephalitis, keratitis, secondary bacterial infections, and deep-tissue monkeypox abscess [8,10,11].

Skin lesions are reported in the vast majority of MPX cases and constitute the predominant feature that leads to MPXV infection diagnosis. A wide spectrum of skin lesions has been described to be associated with MPX, including macular, pustular, vesicular, and crusted lesions, as well as lesions in multiple phases presenting simultaneously [8,9]. Mucosal involvement has also been described in up to 41% of MPX patients, including the anorectal, oropharyngeal, and conjunctival mucosa [9]. More atypical presentations such as single lesions, whitlows, macular eruptions, or kissing lesion configurations have also been described [11,12].

This report aims to describe the number and anatomical localization of lesions from all confirmed MPX patients attending our department with a compilation of clinical images.

## 2. Materials and Methods

In this retrospective, observational analysis, we reviewed the demographic and clinical characteristics of all individuals with confirmed MPXV infection diagnosed by the Department of Dermatology of Centro Hospitalar Universitário Lisboa Norte until 15 July of 2022. The lesions’ number, localization, and characteristics were recorded at diagnosis. The diagnosis was confirmed with swab samples from the skin or mucosa lesions (suspected lesions at the clinician’s discretion). Laboratory diagnosis was performed at the Portuguese National Sexually Transmitted Infections (STI) Reference Centre with PCR in real-time based on detecting the orthopoxvirus genus gene rpo18, followed by Sanger sequencing of PCR products from the lesions, as previously described [13].

## 3. Results

A total of 47 patients were included (Table 1), all men, with an average age of 35.1 years old. Regarding sexual orientation, most patients identified as men who have sex with men (MSM) (83.0%, *n* = 39), followed by heterosexual (8.5%; *n* = 4), bisexual (6.4%; *n* = 3), and data was missing for one patient. A total of 44.7% (*n* = 21) patients were people living with HIV, and 55.3% (*n* = 26) had previously unknown or negative HIV status.

**Figure 1:**
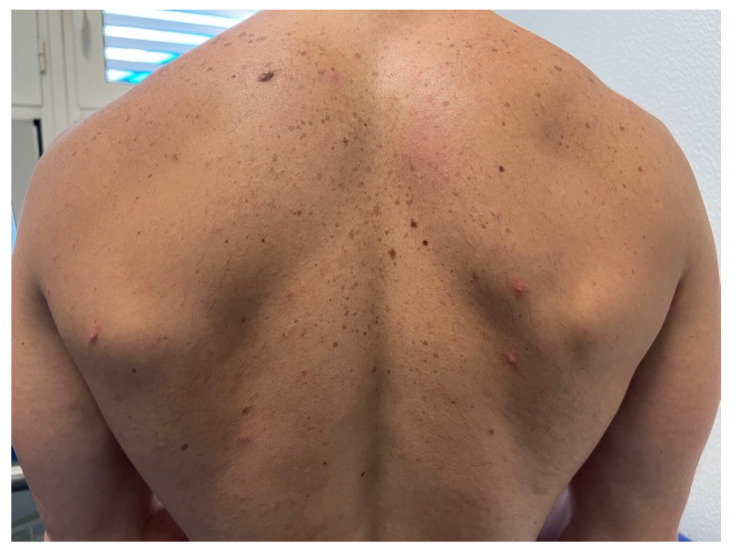
Example of MPX papules in the trunk.

**Figure 2:**
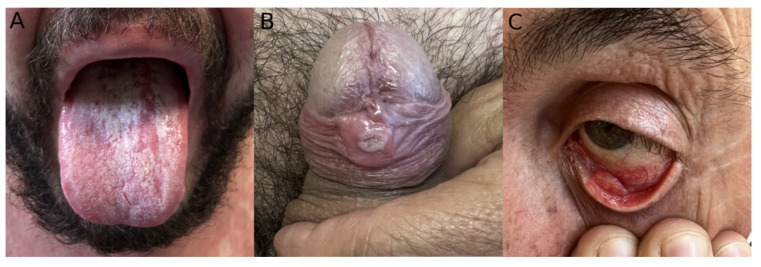
Example of MPX mucosal involvement. (**A**) Oral ulcerations. (**B**) Umbilicated papule in the genital mucosa. (**C**) Palpebral conjunctiva ulceration.

Skin lesions were noted in all patients (Figure 1). The most common anatomical affected sites were the genital area (63.8%; *n* = 30) (Figure 2A), the anorectal area (46.8%, *n* = 22); the trunk, arms or legs (44.7%; *n* = 21); and the face (27.7%; *n* = 13) (Figure S1). One person presented with a singular genital ulcer. Of the total patients observed, only two had more than 20 lesions at the time of observation, one of them in a disseminated pattern. Anorectal mucosa involvement was reported to be associated with anorectal pain, proctitis, or anal mucus discharge (or a combination of these symptoms). Extra anogenital mucosal involvement was present in three patients with oral erosions (Figure 2B) and one with conjunctival mucosal involvement (Figure 2C).

**Figure 3:**
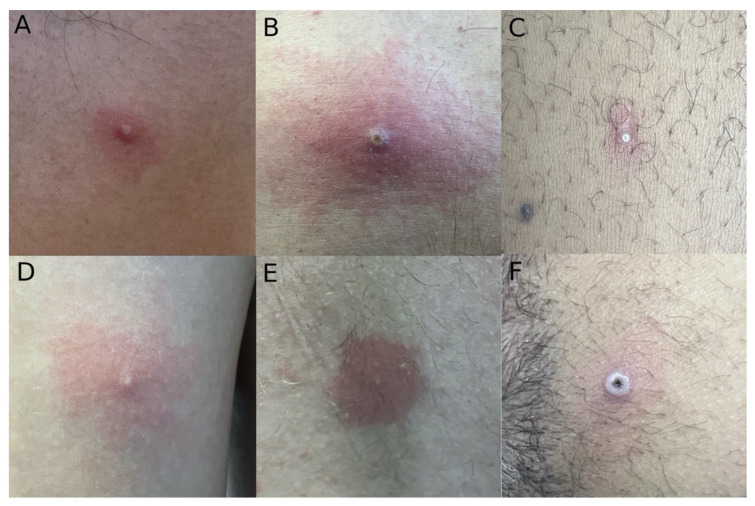
MPX typical skin lesions. (**A**) White papule with an erythematous halo. (**B**) Whitish papule with a necrotic center in an erythematous background. (**C**) Millimetric white papule with an erythematous halo. (**D**) White papule with erythematosus background. (**E**) Millimetric whitish papule with a well-defined erythematous halo. (**F**) White umbilicated papule with a necrotic center.

Typical skin findings included erythematous papules and whitish papules (Figure 3 and Figure S2). Umbilicated papules were also common, some with a necrotic center and an elevated whitish border (Figure 3F). Oral mucosa ulcers presented with well-defined borders and a clean base (Figure 2B). Most patients had lesions in multiple phases presenting simultaneously.

Common systemic features during the course of the illness included lymphadenopathy (68.1%), myalgia (55.3%), fever (53.2%), and headache (48.9%); symptoms that frequently preceded the cutaneous lesions. No patient required monkeypox-specific treatment. Most cases were mild and self-limited, and there were no deaths. One patient required hospital admission in the setting of MPX and acute HIV coinfection. The most common complications were pain and soft-tissue bacterial superinfection. The most reported post-acute complications were cicatricial lesions on the skin and post-inflammatory hyperpigmentation.

## 4. Discussion

MPX is rapidly spreading worldwide in an unprecedented outbreak. This report provides an expansion on the clinical presentations of which health workers should be aware to diagnose MPX in a timely fashion.

Differential diagnoses of MPX lesions include other ulcerative STI—genital herpes, syphilis or chancroid—particularly when the genital and anal areas are the most affected sites [14]. However, a diagnostic clue is the absence of a typical herpetiform figuration, where vesicles are frequently seen packed closely together in genital herpes. Different from syphilis chancre, these lesions are painful, and unlike chancroid, they appear as papules that later progress to an umbilicated and ulcerative form. The papular lesions can also suggest a poxviridae infection, as seen in molluscum contagiosum (MC); or a Varicella-zoster virus (VZV) infection [15,16]. However, MC lesions usually have an evolution more extended in time and are not accompanied by lymphadenopathy; and VZV is characterized not only by papules but also by vesicles and pustules in a dermatomal distribution.

Our findings confirm the previously described skin lesions associated with monkeypox are essential for a clinical diagnosis. All of our patients had skin lesions, which can be explained by the referral to our dermatology department of patients seen with skin alterations in the emergency department. Although classicaly described as a papular, vesicular, and pustular skin eruption, our data show that erythematous papules are the most commonly found lesion in MPX patients [8]. We consider that the previously described vesicles and pustules are, in fact, pseudo-pustules, with no roof to scrap and not filled with purulent material. This is also supported by the histological description of MPX lesions as keratinocytic debris and inflammatory cells but not liquid [17]. Moreover, as other authors have described, the typical dermatological MPX lesion is a papule, which can have different presentations, simultaneously or not in the same patient, with an erythematous base, umbilicated with a whitish border or a necrotic center (or a combination of these) [11].

The lesions distribution in our sample is according to previously described MPX characteristics in this outbreak [9,11], with most lesions localized in areas of close contact during sexual intercourse. In our sample the most common anatomical affected site was the genital area, in 63.8% (*n* = 30) patients, a finding also described in large MPX patient series with prevalence ranging from 53% to 68% [9,11]. The second most common area with lesions was the anorectal zone in 46.8% (*n* = 22) patients. A singular genital ulcer was observed in one patient (2.1%), a less common presentation that can be found in up to 11% of MPX patients [11], once again reinstating the need to include MPX in the differential diagnosis of patients presenting with a singular anogenital ulcerated lesion. Two patients (4.3%) had more than 20 lesions at the time of observation, a less frequently encountered presentation that can affect up to 11% of MPX patients [9]. Both the distribution of lesions and the population characteristics (all men and most MSM) support the most commonly accepted idea that MPX seems to have an STI-like spreading pattern [9,11,14].

Clinical outcomes in this case series were reassuring, as most cases were mild, and only one patient required hospital admission, which has already been described in the literature [12]. Common systemic features encountered included lymphadenopathy (68.1%), myalgia (55.3%), fever (53.2%), and headache (48.9%); results that are in line with the most common symptom triad described in MPX patients besides skin/mucosal lesions: lymphadenopathy, fever and asthenia/lethargy [9,11]. Patients with severe disease or at risk for severe disease include those with mucosal or significant anogenital involvement [18]. These patients appear to be more symptomatic, with painful and eroded lesions, and have a higher number of complications, particularly superimposed bacterial skin infection [18].

Correct identification of MPX skin and mucosal lesions is fundamental for a timely MPX diagnosis in order to provide appropriate management of these patients, prevent further dissemination, and decrease the risk for MPX complications, ensuring less worldwide morbidity.

## 5. Conclusions

Our findings aim at improving MPX case recognition and definition, infection control policies, contact tracing, and future management. Long-term clinical data will help to describe risk factors associated with sequelae, complications of infection, and outcomes in patients living with HIV or presenting with other comorbidities. Further studies are needed to support MPX management and describe the wide clinical presentations occurring during this outbreak in order to decrease onward virus transmission, making use of strategies such as health promotion, preventative or post-exposure vaccination, and antiviral therapy.

## Figures and Tables

**Table 1:** Study population.

Age	M (SD)
Years of age	35.1 (8.7)
**Gender**	***n*** **(%)**
Men	47 (100)
**Sexual orientation**	***n*** **(%)**
MSM	39 (83.0)
Heterosexual	4 (8.5)
Bisexual	3 (6.4)
Missing data	1 (2.1)
**HIV status**	***n*** **(%)**
Positive	21 (44.7)
Unknown/Negative	28 (55.3)
**Number of sexual partners in the last six months**	***n*** **(%)**
<10	38 (80.8)
>10	6 (12.7)
Missing data	3 (6.4)

MSM: men who have sex with men.

## Data Availability

Not applicable.

## Supplementary Materials

The following supporting information can be downloaded at: https://www.mdpi.com/article/10.3390/idr14050077/s1, Figure S1: Examples of MPX skin involvement. (A) Whitish papule with necrotic center and an erythematous halo on the gluteal area. (B) Erythematous papules on the neck. (C) Erythematous papules on the chest. (D) Whitish perianal papules. (E) Whitish papule on the thigh. (F) Erythematous papules on the palm, Figure S2: MPX typical skin lesions. (A) Whitish papules with an erythematous halo. (B) Confluent papules with necrotic center and whitish borders in an erythematous background. (C) Whitish papules, some with necrotic center. (D) Whitish umbilicated papules with necrotic center.
