# Supplementary material for: Monkeypox Diagnosis by Cutaneous and Mucosal Findings
Source: Infect Dis Rep. 2022 Sep 27;14(5):759–64. doi: 10.3390/idr14050077 (PMC9602055; doi:10.3390/idr14050077)
Supplement: Supplementary file 1 [file idr-14-00077-s001.zip › idr-1892049-supplementary.pdf]

Supplementary Materials

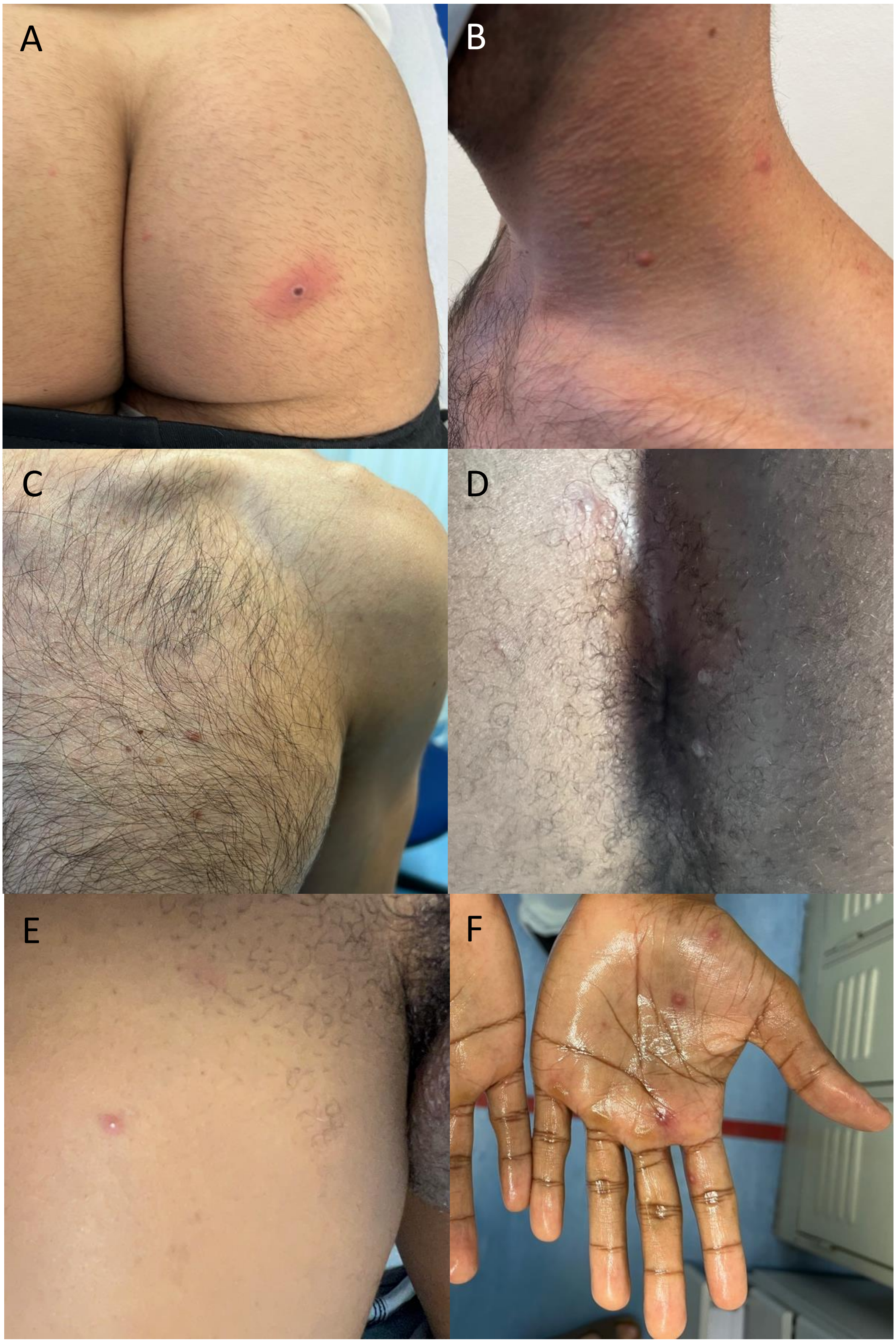

Figure S1. Examples of MPX skin involvement. (A) Whitish papule with necrotic center and an erythematous halo on the gluteal area. (B) Erythematous papules on the neck. (C) Erythematous papules on the chest. (D) Whitish perianal papules. (E) Whitish papule on the thigh. (F) Erythematous papules on the palm.

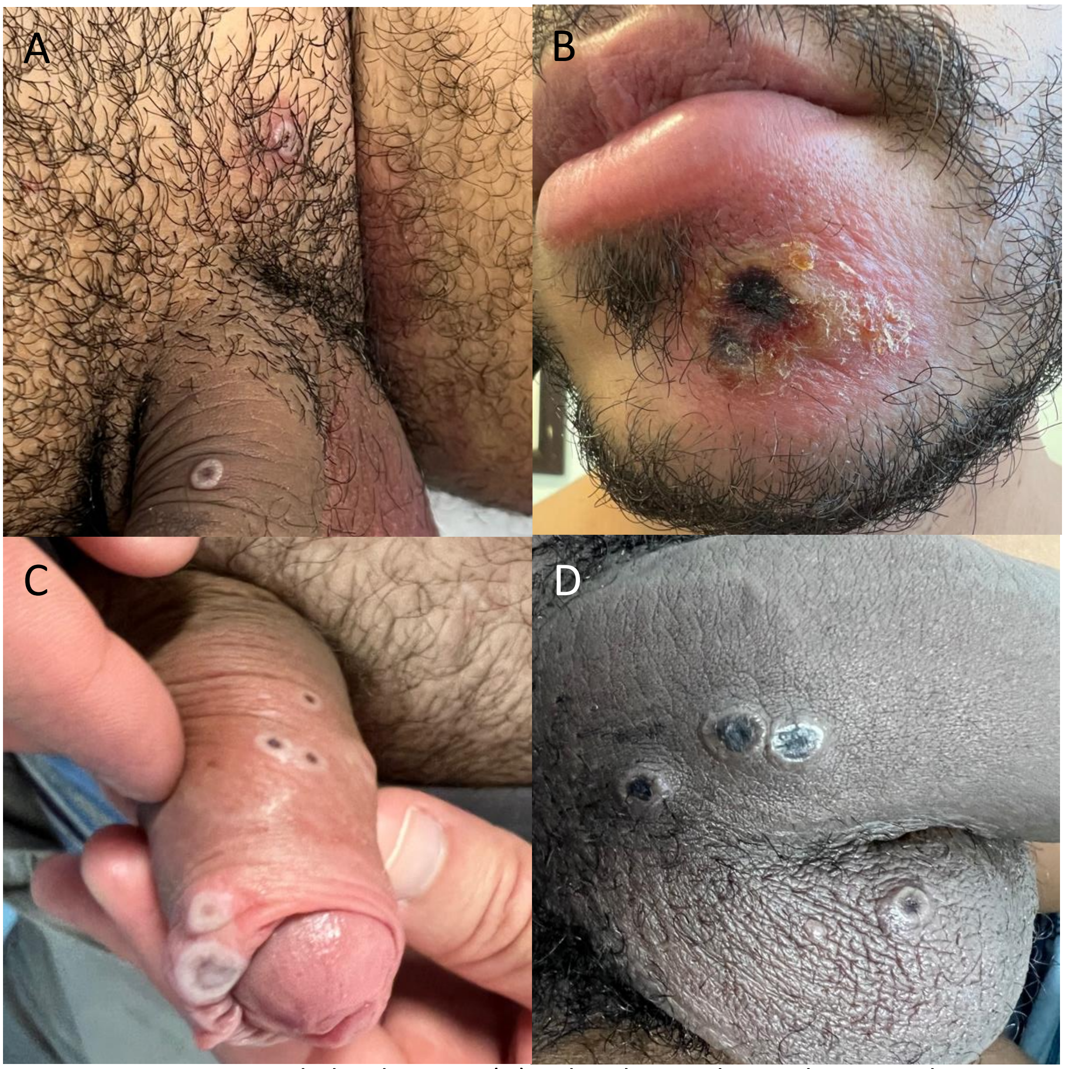

Figure S2. MPX typical skin lesions. (A) Whitish papules with an erythematous halo. (B) Confluent papules with necrotic center and whitish borders in an erythematous background. (C) Whitish papules, some with necrotic center. (D) Whitish umbilicated papules with necrotic center.
